# Prioritizing genes responsible for host resistance to influenza using network approaches

**DOI:** 10.1186/1471-2164-14-816

**Published:** 2013-11-21

**Authors:** Suying Bao, Xueya Zhou, Liangcai Zhang, Jie Zhou, Kelvin Kai-Wang To, Binbin Wang, Liqiu Wang, Xuegong Zhang, You-Qiang Song

**Affiliations:** Department of Biochemistry, The University of Hong Kong, Hong Kong, China; Bioinformatics Division and Center for Synthetic and Systems Biology, TNLIST, MOE Key Lab of Bioinformatics / Department of Automation, Tsinghua University, Beijing, China; Department of Biophysics, College of Bioinformatics Science and Technology, Harbin Medical University, Harbin, China; Department of Microbiology, The University of Hong Kong, Hong Kong, China; Carol Yu Centre for Infection, The University of Hong Kong, Hong Kong, China; National Research Institute for Family Planning, Beijing, China; Department of Mechanical Engineering, The University of Hong Kong, Hong Kong, China; HKU-Zhejiang Institute of Research and Innovation (HKU-ZIRI), Linan, Zhejiang, 311100 China; Center for Genome Science, The University of Hong Kong, Hong Kong, China

## Abstract

**Background:**

The genetic make-up of humans and other mammals (such as mice) affects their resistance to influenza virus infection. Considering the complexity and moral issues associated with experiments on human subjects, we have only acquired partial knowledge regarding the underlying molecular mechanisms. Although influenza resistance in inbred mice has been mapped to several quantitative trait loci (QTLs), which have greatly narrowed down the search for host resistance genes, only few underlying genes have been identified.

**Results:**

To prioritize a list of promising candidates for future functional investigation, we applied network-based approaches to leverage the information of known resistance genes and the expression profiles contrasting susceptible and resistant mouse strains. The significance of top-ranked genes was supported by different lines of evidence from independent genetic associations, QTL studies, RNA interference (RNAi) screenings, and gene expression analysis. Further data mining on the prioritized genes revealed the functions of two pathways mediated by tumor necrosis factor (TNF): apoptosis and TNF receptor-2 signaling pathways. We suggested that the delicate balance between TNF’s pro-survival and apoptotic effects may affect hosts’ conditions after influenza virus infection.

**Conclusions:**

This study considerably cuts down the list of candidate genes responsible for host resistance to influenza and proposed novel pathways and mechanisms. Our study also demonstrated the efficacy of network-based methods in prioritizing genes for complex traits.

**Electronic supplementary material:**

The online version of this article (doi:10.1186/1471-2164-14-816) contains supplementary material, which is available to authorized users.

## Background

Influenza is a highly contagious, seasonal respiratory illness caused by the influenza virus. The progression and outcome of pathogenic infections are influenced by host genetic factors [1–7]. Further studies showed that this finding may also hold true for influenza A virus infection [8–12]. Thus host genetic factors should be identified to gain insights into the molecular mechanisms underlying host resistance and accelerate the development of new therapeutic regimes for patients. Several genome-wide quantitative trait locus (QTL) mapping studies have been conducted using different mouse strains to identify host genetic factors that contribute to the resistance to influenza virus infection [10, 13–16]. The identified QTLs have greatly narrowed the scope of genetic factors from the whole genome to a set of genomic intervals. However, identifying the underlying genes from a large number of candidates within these regions remains a challenge. In this study, *in silico* approaches were used to prioritize a list of the most promising candidate genes from these QTL regions for future investigations.

The basic idea for most computational gene prioritization is that for a heritable trait with genetic heterogeneity, different trait-related genes should show similarities with one another based on some particular measure. Assuming that the known disease genes (termed “seed genes” or “seeds”) represent all of the genes responsible for a specific disease, then the unknown disease genes can potentially be distinguished from other candidates based on their similarities to the seeds (so called “seed-based” strategy). With the accumulation of high-throughput protein-protein interaction data, network-based similarity measures were demonstrated to be effective in prioritizing human disease genes using the seed-based strategy [17]. We first showed that a scoring method based on these measures could have reasonable power to predict known host resistance genes. However, the “seed-based” methods have several drawbacks stemming from an inherent limitation: these methods rely on known disease genes, which are incomplete in some studies and may introduce considerable bias. Meanwhile, many microarray experiments comparing the gene expression profiles of cases and controls have been performed. These studies contained rich information regarding trait-related genetics, but the information has not been fully exploited. Previous studies showed that disease genes are often surrounded by differentially expressed neighbors in a gene network, but not necessarily highly differentially expressed themselves [18, 19]. We further demonstrated that host resistance genes also share this property in a protein association network. Several scoring approaches using DE levels of network neighbors were evaluated to prioritize known host resistance genes. Our evaluation suggested that DE-based methods could also effectively prioritize the genes responsible for host resistance to influenza.

By applying both strategies to prioritize genes within mouse QTLs associated with host resistance to influenza, we identified functional relevant genes that were supported by multiple lines of evidence from previous studies. A list of promising candidate genes strongly supported by literatures was totally missed when seed-based methods were used. Using the DE-based method, we were able to identify these genes. This result indicated that the DE-based strategy can complement the seed-based strategy to obtain novel candidates without the influence of limited knowledge. In addition, evidence-supported genes were significantly enriched in top-ranked genes prioritized by both seed- and DE-based strategies. Hence, DE-based strategy can also enhance the credibility of the inference of a candidate’s role in the pathogenesis of a disease. The results of functional enrichment analysis further showed that genes prioritized by both strategies revealed several biological processes that may exert critical functions in influencing host outcomes after influenza virus infection. In summary, our results suggested that the DE-based strategy can provide additional benefits and reduce the bias from a limited set of known disease genes. These results can also enhance our understanding of the pathological pathways of influenza.

## Results and discussion

**Figure 1:**
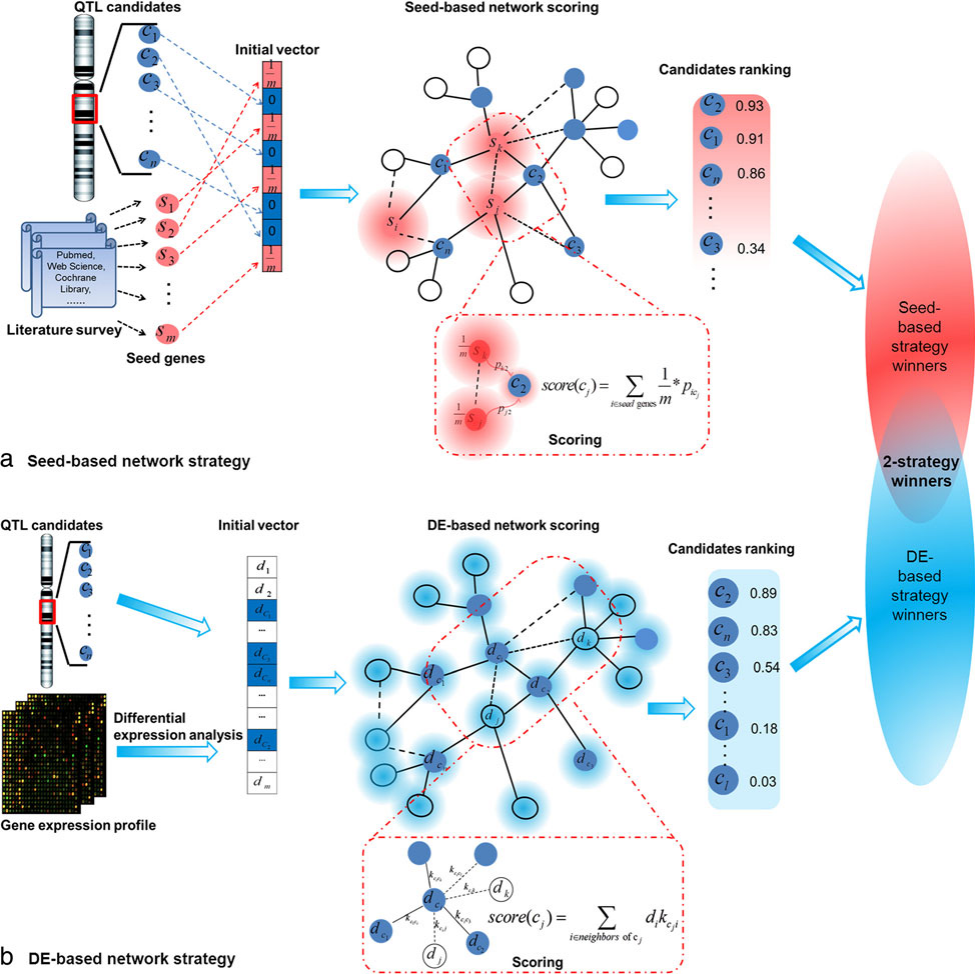
**Overview of the network approaches based on seed genes and differential expression.** The gene network is constructed from STRING database and represented by an undirected graph consisting of nodes (genes) and weighed edges (links between gene pairs with associated scores). **(a)** For the seed-based strategy, the score vector for all seeds and other genes within the genome is initialized with the entries corresponding to the seed genes assigned with equal scores whose sum is equal to 1. The vector is iteratively updated by a random walk process over the network until it reaches convergence. Candidate genes are ranked by their scores in the converged vector, which can be interpreted as the steady-state probabilities of staying at the nodes representing the candidate genes. A high probability for the candidate corresponds to a higher similarity to the seeds. As a computationally efficient alternative, a heat kernel diffusion matrix can be used to approximate the distances between all pairs of genes. The candidate genes are then scored according to their average distances to the seeds based on the kernel matrix. **(b)** The DE-based method does not rely on the definition of seeds but uses a trait-related microarray expression profile to obtain the DE levels of the genes. DE levels were then mapped onto the network. For each candidate gene, the score is calculated as a weighted average of the DE levels of the gene and its network neighbors with the weights derived from the network distances between genes. In this study, the candidate genes within each QTL were scored using two different strategies, and the top 10**%** ranked by each method was chosen as prioritized genes (winners).

The overall prioritization strategy was shown in Figure 1. Each candidate gene within the QTL intervals associated with host resistance to influenza was scored using seed- (Figure 1a) and DE-based strategies (Figure 1b). We used the gene association network compiled by the STRING database (version 9) [20] to derive the similarity measures and network neighbors. Top 10% of the genes within each QTL region ranked by either seed- or DE-based scoring strategy were considered as prioritized. All of the prioritized genes were then subject to systematic literature survey and gene set enrichment analysis.

### Optimizing the network similarity measures for seed-based methods

**Figure 2:**
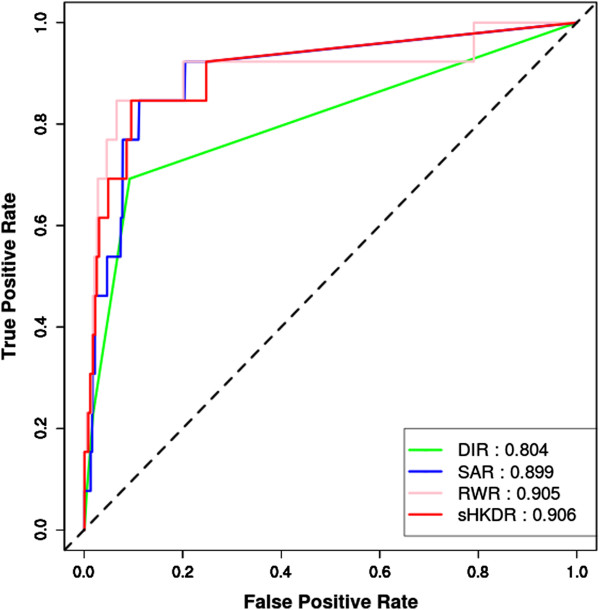
**Performance evaluation of seed-based network strategy.** The ROC curves of the seed-based methods in LOOCV test on known host resistance genes. Four different methods (DIR, SAR, RWR, and sHKDR) as described in the main text were compared. The prioritization performance can be measured as AUC presented next to each method.

For the first seed-based scoring strategy (Figure 1a), 14 genes were collected as seeds that harbor variants (either natural polymorphisms or knockouts in model organisms) associated with the traits related to host resistance after influenza virus infection (Table 1). To best capture the relationships among host resistance genes, we evaluated the performance of several different network similarity measures: direct interaction ranking (DIR), STRING association ranking (SAR), random walk with restart (RWR), and seed-based heat kernel diffusion ranking (sHKDR). The DIR measure for a gene corresponds to the number of direct interactions (above a specific threshold) with seeds; SAR is the sum of direct interaction scores. More sophisticated methods were also applied. One method uses the arrival probability in the steady state of random walks with restart from seeds in the gene network (RWR); the other measures the average distances to the seeds represented by a diffusion heat kernel matrix (sHKDR). The mathematical details of these scoring methods can be found in Additional file 1. To evaluate the model performance, we randomly chose 99 genes as background for each seed. Each seed and its corresponding random background were then scored by the model built from the remaining seed genes. This step is called the leave-one-out cross validation (LOOCV) test (Materials and methods). The model performance can be reflected by the ranks of the seed genes over the background and quantified as the area under the curve (AUC) of the receiver operating characteristic (ROC; Figure 2) [21]. The model parameters in sHKDR (diffusion factor *β* and iteration time *m*) were tuned to optimize the performance (Additional file 1: Table S1). Figure 2 shows that RWR (AUC = 0.905) and sHKDR (AUC = 0.906), both of which consider indirect interactions, exhibit similar performances and outperform SAR (AUC = 0.899) and DIR (AUC = 0.804) in terms of AUC values. Therefore, we chose RWR and sHKDR as the measures for the seed-based scoring strategy. Furthermore, the ROC curves also suggested that known resistance genes can be ranked at the top 10%in the simulated candidate sets among 85% of total prioritization processes using RWR, which is superior to other measures at the same ranking percentage.

**Table 1:** **The collection of 14 known host resistance genes**

Entrez ID	Gene symbol	Gene description	Mouse ortholog	Cytoband	Supporting evidence
4599	*MX1*	myxovirus (influenza virus) resistance 1	*Mx1, Mx2*	21q22.3	Mouse strains homozygous for *Mx* null allele fail to synthesize Mx protein and are influenza virus susceptible [22].
9437	*NCR1*	natural cytotoxicity triggering receptor 1	*Ncr1*	19q13.42	*Ncr1* ^-/-^ 129/Sv and C57BL/6 mice were lethal after influenza virus infection [23].
1234	*CCR5*	chemokine (C-C motif) receptor 5	*Ccr5*	3p21.31	Deaths among *Ccr5* ^-/-^ mice increase after infection with influenza A virus [22]. A large proportion of heterozygosity for the *CCR5Δ*32 allele among white patients with severe disease was also found [24].
114548	*NLRP3*	NLR family, pyrin domain containing 3	*Nlrp3*	1q44	Mice lacking *Nlrp3* exhibited dramatically increased mortality and a reduced immune response after exposure to the influenza virus [25]. Gene polymorphisms in the *NALP3* inflammasome are associated with interleukin-1 production and severe inflammation in human [26].
3105	*HLA-A*	major histocompatibility complex, class I, A	*H2-D1*	6p21.3	The magnitude and specificity of influenza A virus-specific cytotoxic T-lymphocyte responses in humans is associated with the HLA-A and -B phenotypes [27].
3106	*HLA-B*	major histocompatibility complex, class I, B			
2212	*FCGR2A*	Fc fragment of IgG, low affinity IIa, receptor (CD32)	*Fcgr3*	1q23	rs1801274 on *FCGR2A* is significantly (p < 0.0001, OR = 2.68, 95% CI: 1.69-4.25) associated with sever pneumonia after A/H1N1 infection in human [28].
84268	*RPAIN*	RPA interacting protein	*Rpain*	17p13.2	rs8070740 on *RPAIN* is significantly (p < 0.0001, OR = 2.67, 95% CI: 1.63-4.39) associated with sever pneumonia after A/H1N1 infection in human [28].
3456	*IFNB1*	interferon, beta 1, fibroblast	*Ifnb1*	9p21	*IFN*-*β*-deficient mice carrying functional *Mx1* alleles showed 20-fold lower in the 50% lethal dose of H7N7; and also substantially reduced resistance to H1N1 infection [29].
3586	*IL10*	interleukin 10	*Il10*	1q31-q32	A promoter polymorphism conferred a significantly decreased risk of adverse response to inactivated influenza vaccine [30].
708	*C1QBP*	complement component 1, q subcomponent binding protein	*C1qbp*	17p13.3	rs3786054 on *C1QBP* is significantly (p < 0.0001, OR = 3.13, 95% CI: 1.89-5.17 ) associated with sever pneumonia after A/H1N1 infection in human [28].
3811	*KIR3DL1*	killer cell immunoglobulin-like receptor, three domains, long cytoplasmic tail, 1	*Kir3dl1*	19q13.4	KIR3DL1/S1 and 2DL1 ligand-negative pairs were enriched among H1N1 ICU cases [31].
3803	*KIR2DL2*	killer cell immunoglobulin-like receptor, two domains, long cytoplasmic tail, 2	*Kir3dl2*	19q13.4	KIR2DL2/L3 ligand-positive pairs were enriched among H1N1 ICU cases [31].
10410	*IFITM3*	interferon induced transmembrane protein 3	*Ifitm3*	11p15.5	Mice lacking *Ifitm3* display fulminant viral pneumonia when challenged with a normally low-pathogenicity influenza virus. A statistically significant number of hospitalized subjects were also shown enrichment for a minor *IFITM3* allele that alters a splice acceptor site [32].

### Evaluating the performance of DE-based network strategy

**Figure 3:**
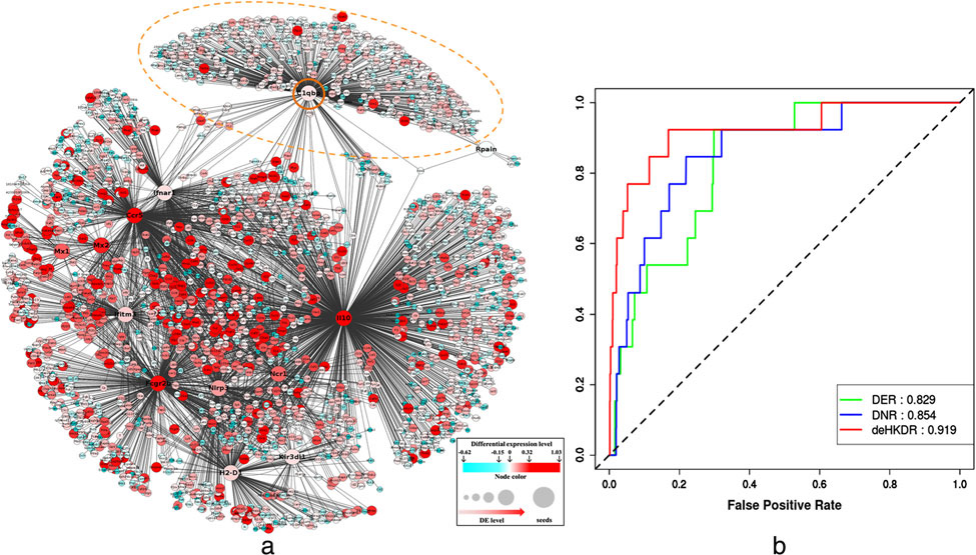
**Empirical survey and performance evaluation of DE-based network strategy. (a)** The known influenza host resistance genes are surrounded by differentially expressed genes between resistant and susceptible mouse strains. To visualize the gene expression levels within a network context, a sub-network consisting of only the seed genes and their directly linked neighbors in the STRING database was extracted and visualized using Cytoscape [34] under the edge-weighed spring embedded layout. The distances between seeds and their neighbors were set proportional to their interaction scores. Differential expression levels between resistance and susceptible mouse strains are mapped to the size and color shade of each node. The significant differentially expressed genes were highlighted by unifying the colors of genes with DE levels that ranked at the top 5% (DE level ≥ 0.32) among the whole genome in red and the genes with DE levels that ranked at the bottom 5% (DE level ≤ -0.15) in blue (as illustrated in the inset). All seed genes are highlighted using the same node size and bold fonts of their names. **(b)** The ROC curves of DE-based methods in the validation test on known host resistance genes. Three methods (DER, DNR, and deHKDR) as described in the main text were compared. The performance measured as AUC is shown next to the name of each method.

To apply the DE-based network strategy, we empirically surveyed the DE levels of 14 known host resistance genes and their neighborhoods in the STRING network. We first obtained the whole-genome expression profiles of 44 pre-Collaborative Cross (CC) mice after being infected by influenza virus (GSE30506 [33]). The DE level was measured as the log2 ratios of the mean expression values between 26 susceptible strains and 18 resistant strains. A sub-network comprising all of the seed genes and their interacting neighbors was extracted from the STRING network (Figure 3a). The node sizes and shades of colors were used to represent the DE levels. We found that most of the seeds here were surrounded by differentially expressed neighbors. Some of the seeds, such as *C1qbp*, which is not directly linked to other seed genes, may lose their priority when seed-based methods were used (highlighted by a yellow circle; the sub-network of this gene and its neighbors are shown in Additional file 1: Figure S1a). Some of the seed genes, such as *H2-D1*, *Ifnar1*, and *Ifitm3*, were not highly differentially expressed, but these genes were surrounded by highly differentially expressed neighbors in the network (Additional file 1: Figure S1 b-d). These observations suggested the feasibility of incorporating the DE levels of network neighbors to prioritize host resistance genes.

To quantitatively assess the hypothesis that the genes responsible for host resistance to influenza virus infection are surrounded by network neighbors differentially expressed between resistant and susceptible mouse strains, we evaluated three DE-based scoring methods to prioritize known resistance genes. These methods include: Differential Expression Ranking (DER, scoring each gene based on its own DE level), Direct Neighborhood Ranking (DNR, weighted sum of the gene’s own DE level and the average of all direct neighbors), and DE-based HKDR (deHKDR, weighted sum of the gene’s own DE level and the weighted average of direct and indirect neighbors based on heat kernel diffusion ranking; Materials and methods, Additional file 1). The performances of DE-based methods were also assessed by the ranks of seeds relative to the randomly sampled genes and quantified as the AUC of ROC. In contrast to the LOOCV used for seed-based methods, seeds and background genes were all scored using DE-based methods.

The required parameters (steady factor *α* in DNR; *β* and *m* in deHKDR) were tuned to maximize the AUC for each method (Additional file 1: Tables S2 and S3). In Figure 3b, the method that aggregated weighted DE levels of all surrounding genes (deHKDR, AUC = 0.919) showed better performance than the ranking methods that relied on DE alone (AUC = 0.829 for DER) or the method that only considered the unweighted DE levels of direct neighbors (AUC = 0.854 for DNR). The performance of deHKDR was comparable to that of the seed-based methods (RWR and sHKDR) in terms of AUC. The ROC curve also suggested that the known resistance gene can be found among the top 10% of the scored genes with probability higher than 0.75. These results indicated that the known resistance genes were possibly surrounded by differentially expressed neighbors; therefore, DE-based scoring methods can be applied to prioritize host resistance genes.

### Prioritizing candidate genes within mouse QTLs

**Figure 4:**
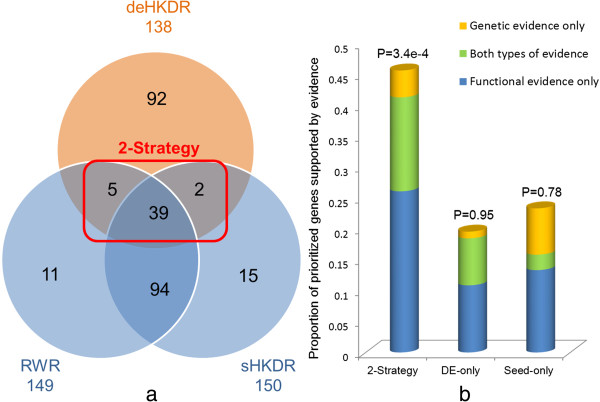
**An overview of the prioritized genes from mouse QTLs. (a)** A total of 258 genes (winners) were ranked at the top 10**%** in each QTL region by the seed- (RWR, sHKDR) or DE-based method (deHKDR). The numbers of winners identified by one, two, or all three methods are shown in a Venn diagram. The winners identified by at least one of the seed-based methods and by the DE-based method were termed 2-strategy winners. The remaining winners (identified by the seed-based methods only or by DE-based method only) were termed single-strategy winners. **(b)** 2-strategy winners are better supported by the genetic or functional evidence compared with single-strategy winners. Each set of winners(2-strategy winners, DE-only winners, seed-only winners) was annotated by genetic evidence and functional evidence. The proportion of winners supported by one class of evidence or both was plotted as a stacked cylinder. One-tailed hypergeometric test was used to determine the enrichment significance of the supported winners (either supported by genetic or functional evidences) in a winner set, given all prioritized winners as background. P values were annotated above the corresponding cylinders.

We applied seed- and DE-based strategies to score and rank the candidate genes in 17 reported mouse QTLs (Table 2). We aimed to use a mouse model to inform human diseases; thus only conserved mouse genes with human orthologs were selected as candidates (Materials and methods). For each QTL region, the candidate genes ranked at the top 10% by each method (RWR, sHKDR, and deHKDR) were considered as prioritized genes for a specific method. The number of the genes prioritized using the three methods was shown as a Venn diagram in Figure 4a (detailed functional annotations are given in Additional file 2: Table S4). Among the 258 genes, 46 were prioritized by at least one seed-based method (RWR or sHKDR) and a DE-based method (deHKDR); these genes were then termed as 2-strategy winners (Figure 4a). To systematically collect supporting evidence for prioritized genes, we searched the following four types of studies that are related to host resistance or response to influenza virus infection (Materials and methods): genetic association studies [22, 27, 35–41], QTL studies [10, 14–16, 33], RNA interference (RNAi) screenings [42–46], and microarray gene expression profiles [47–49]. Among the top-ranked genes, 12 of them were reporeted to harbor polymorphisms associated with the outcome related to influenza infection, including *ACE*[50], *HLA-DQB1*[35], *LTA*, *TNF*[36], *PSMB9*[37], *EIF2AK2*[38], *C5*[39, 40], *IL1RN*[41], *IL12RB2*[41], *MX1*[22], *HLA-A*, and *HLA-B*[27], which strongly support their roles as host genetic factors. *MX1*, *HLA-A*, and *HLA-B* were the seeds used for the seed-based strategy; however, these genes, except for *HLA-A*, were also identified using the DE-based strategy. Another 64 genes are considered as promising candidates responsible for host resistance by QTL studies or genes related to host response to influenza virus infection by RNAi screenings or gene expression analysis (Additional file 2: Table S4). Other literature supporting for the function of a gene in host resistance or response to influenza infection were listed in the last two columns of Additional file 2: Table S4. Top-ranked genes supported by multiple types of studies (genetic association, QTL, RNAi, or expression studies), with a total of 19 genes, are listed in Table 3. Among these genes, seven were identified by both seed- and DE-based strategies; seven were specifically prioritized by the DE-based strategy; the remaining genes were identified by the seed-based strategy (Table 3). This observation suggested that the DE-based strategy, using a completely different prioritization mechanism from seed-based strategy, can complement the seed-based strategy to identify promising disease genes.

**Table 2:** **QTL studies for candidate genes collection**

Study^*^	QTL regions^†^	Influenza virus	Mouse strains
Toth et al., 1999 [13]	chr6:48676555-75397704	H3N2	CXB
Boon et al., 2009 [10]	chr2:33–52 Mb;	H5N1	BXD
	chr7:107–121 Mb;		
	chr11:101–107 Mb;		
	chr15:51–57 Mb;		
	chr17:68–84 Mb		
Nedelko et al., 2012 [15]	chr2:56–68 Mb;	H1N1	BXD
	chr5:140–153 Mb;		
	chr16:64–78 Mb;		
	chr17:30–44 Mb;		
	chr19:37–45 Mb		
Boivin et al., 2012 [14]	chr2:24–38 Mb;	H3N2	AcB
	chr17:37–48 Mb		
Ferris et al., 2013 [16]	chr1:21767867–29085401;	H1N1	preCC
	chr16:97500418–98213493;		
	chr7:89130587–96764352;		
	chr15:77427235-86625488		

^*^The QTL regions were collected from genome-wide scans of phenotypes related to the outcome of influenza virus infection in inbred mouse.

^†^The genomic positions are based on the coordinates of NCBI build 37.

**Table 3:** **Prioritized genes supported by multiple types of studies**

Gene symbol	Gene description	Prioritization method		Supporting source*				Functional annotation and/or literature support
		Seed-based	DE-based	Genet-Assoc	QTL	RNAi	Expr	
*IFI35*	interferon-induced protein 35		+		+	+	+	*Ifi35* can be up-regulated upon exposure to interferon and modulate the cytokine signaling [35]. It also has antiviral properties against bovine foamy virus via inhibiting its replication [41].
*EIF2AK2*	eukaryotic translation initiation factor 2-alpha kinase 2	+	+	+	+	+	+	The encoded protein is a serine/threonine protein kinase that is activated after binding to dsRNA during the course of a viral infection. Mice lacking this gene displayed increased susceptibility to influenza virus infection [38].
*TNF*	tumor necrosis factor (TNF superfamily, member 2)	+	+	+			+	The encoded protein is a multifunctional proinflammatory cytokine, involved in the regulation of a wide spectrum of biological processes including apoptosis. It harbored polymorphisms associated with the severity of the clinical behavior after infection by the pandemic influenza A/H1N1 [36].
*TRIM26*	tripartite motif-containing 26	+			+	+		The encoded protein is a member of the tripartite motif (TRIM) family.
*IFIH1*	interferon induced with helicase C domain 1	+	+		+		+	Innate immune receptor acting as a cytoplasmic sensor of viral nucleic acids and plays a major role in the activation of a cascade of antiviral responses including the induction of type I interferons and proinflammatory cytokines. The *Ifih1* knock-out mice exhibit an impaired response to different viral pathogens [51, 52].
*TAP2*	transporter 2, ATP-binding cassette, sub-family B (MDR/TAP)		+		+		+	Involved in antigen processing and presentation.
*FOLH1*	folate hydrolase (prostate-specific membrane antigen) 1		+		+	+		
*HLA-E*	major histocompatibility complex, class I, E	+			+		+	HLA class I molecules play a central role in the immune system by presenting peptides derived from the endoplasmic reticulum lumen.
*LST1*	leukocyte specific transcript 1		+		+		+	The protein encoded by this gene is a membrane protein that can inhibit the proliferation of lymphocytes. In humans, *LST1* plays a role in the regulation of the immune response to inflammatory diseases [53].
*FAM135A*			+		+	+		
*PLA2G7*	phospholipase A2, group VII (platelet-activating factor acetylhydrolase, plasma)		+		+		+	The encoded protein a secreted enzyme that catalyzes the degradation of platelet-activating factor to biologically inactive products. It harbored genetic polymorphisms associated with imflammatory diseases like atopy and asthma in humans [49].
*TAPBP*	TAP binding protein (tapasin)	+	+		+		+	Involved in the association of MHC class I with TAP and in the assembly of MHC class I with peptide.
*PSMB9*	proteasome (prosome, macropain) subunit, beta type, 9 (large multifunctional peptidase 2, LMP2)	+	+	+		+	+	The proteasome is a multicatalytic proteinase complex. The encoded subunit is involved in antigen processing to generate class I binding peptides. The *LMP2*-mutant mice showed reduced levels of CD8+ T lymphocytes and generated 5- to 6-fold fewer influenza nucleoprotein-specific cytotoxic T lymphocyte precursors [37].
*IL1RN*	interleukin 1 receptor antagonist	+		+		+	+	The encoded protein inhibits the activities of interleukin 1 and modulates a variety of interleukin 1 related immune and inflammatory responses. It harbors genetic polymorphisms significantly related to humoral immune response to inactivated seasonal influenza vaccine [41].
*C5*	complement component 5	+		+	+			The encoded protein is the fifth component of complement, which plays an important role in inflammatory and cell killing processes. The *C5*-deficiency was reported to increase susceptibility to mouse-adapted influenza A virus [39, 40].
*DAXX*	death-domain associated protein		+			+	+	The encoded protein may function to regulate apoptosis. Influenza virus can escape the repressional function of Daxx during infection by binding matrix protein 1 with Daxx [54].
*HLA-DQB1*	major histocompatibility complex, class II, DQ beta 1; similar to major histocompatibility complex, class II, DQ beta 1	+		+	+			HLA-DR7/4,DQB1*0302genotype was significantly associated (OR = 5.15; 95%CI = 1.94, 13.67; p = 0.001) with clinical hyporesponsiveness after trivalent inactivated influenza vaccine [35]
*MX1*	myxovirus (influenza virus) resistance 1, interferon-inducible protein p78 (mouse)	+	+	+	+		+	Mice susceptible to influenza infection harbor large exonic deletions or nonsense mutations in the *Mx1* gene [22]. (seed gene)
*HLA-A*	major histocompatibility complex, class I, A	+		+		+		The magnitude and specificity of influenza A virus-specific cytotoxic T-lymphocyte responses in humans is related to HLA-A and -B phenotype [27]. (seed gene)
*HLA-B*	major histocompatibility complex, class I, B	+	+	+	+	+	+	

*The following sources of supporting evidence were collected for each prioritized gene. Genet-Assoc: literature supporting for the gene’s genetic association with host resistance to influenza infection. QTL: candidate genes identified in the original QTL study with independent evidence (harboring founder variants that were associated with the phenotype; co-localization with a cis-eQTL; etc.). RNAi: host genes important for influenza life circle identified through high-throughput RNAi screens. Expr: host genes robustly up- or down- regulated after influenza virus infection identified from multiple microarray experiments. Detailed supporting evidence for each gene was listed in Additional file 2: Table S4. For more details of QTL, RNAi and expression studies, see Additional file 2: Table S5.

To provide an overview of the functional significance of top-ranked genes from seed- and DE-based strategies or both, we summarized the proportions of the winners supported by particular evidence in each winner set. The four types of supporting sources were catergorized into two classes of evidence: genetic evidence (including genetic association studies and QTL studies) and functional evidence (including RNAi screenings and expression analysis; Materials and Methods). Top-ranked genes specifically identified by the DE-based strategy (DE-only) or the seed-based strategy (seed-only) or the winners prioritized by both strategies (2-strategy) were grouped into three winner sets and mapped to the genes supported by genetic evidence and functional evidence or both (Figure 4b). We used the hypergeometric test to evaluate the statistical significance of observing a specific proportion of the supported winners in a winner set given all prioritized winners as background. A significant increase in the proportions of winners supported by all types of supporting sources was observed in the 2-strategy winner set (>45%) compared with the single-strategy winner set (<25%), with a hypergeometric p-value of 3.4e–4. The proportion of the DE-only winners supported by genetic evidence (approximately 10%) was similar to that of seed-only winners; by comparison, a higher percentage (approximately 20%) of functional evidence was observed among the DE-only winners compared with the seed-only winners (approximately 16%). Although microarray expression data were also used in our DE-based strategy, they are independent of the data used in supporting evidence. This finding suggested that the DE-based strategy can provide additional advantages in identifying promising candidates by fully exploiting the rich information from the microarray expression data.

### Pathways and biological functions revealed by top-ranked genes

**Figure 5:**
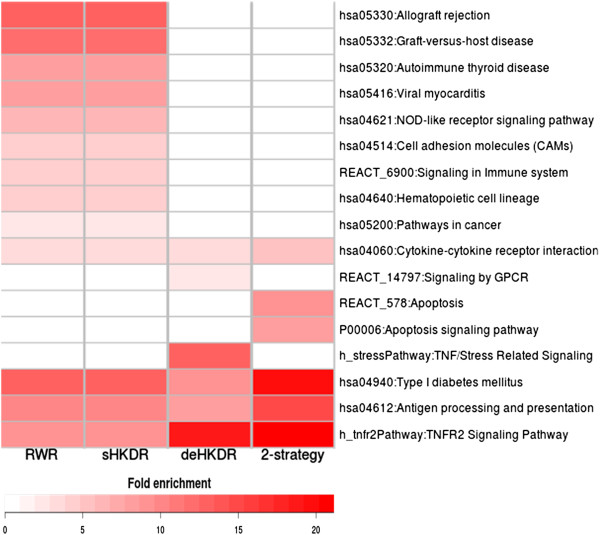
**Pathways enriched by the prioritized genes.** Pathways (KEGG, BioCarta, Reactome) significantly enriched (p < 0.01 and FDR < 0.25) by the winners of each method (RWR, sHKDR, deHKDR) or by 2-strategy winners are shown as a heatmap. The color intensity of each cell represents the fold enrichment of the corresponding winner group for each pathway. Only the significantly enriched pathways for each winner group are shown.

The following gene sets deposited in the DAVID knowledgebase [55] (version 6.7) were used in the functional enrichment analyses: BIOCARTA (http://www.biocarta.com/), KEGG (http://www.genome.jp/kegg/), REACTOME (http://www.genomeknowledge.org/), PANTHER (http://www.pantherdb.org/; including biological process, BP, and molecular function, MF), and Gene Ontology FAT (including BP, MF, and cellular component, CC; Materials and methods). All of the gene sets enriched by each group of winners (2-strategy, deHKDR, sHKDR, or RWR winners) at the nominal significance level of p < 0.01 are shown in Additional file 3. Figure 5 illustrates the pathways significantly enriched (p < 0.01 and false discovery rate, FDR < 0.25) by at least one winner group as a heatmap. The significant results of gene ontology (GO) enrichment (in terms of BP, MF, and CC) are provided in Additional file 1: Figure S2. Figure 5 further shows that the genes prioritized by seed-based methods were more enriched in immune-related pathways (e.g., allograft rejection, NOD-like receptor signaling pathway, and signaling in immune system) compared with those prioritized by the DE-based method. It may reflect the inherent bias of seed-based method: neighboring genes in the STRING network tended to share the same pathways, and seed genes were mostly immune related, so we expected to see winners of seed-based methods to enrich in general immune related pathways. Alternatively, shared gene with other immune related processes can be interpreted as shared genetic causes (pleiotropy) of immune related phenotypes. The genes prioritized by the DE-based method specifically revealed two pathways: TNF/stress-related signaling (p = 2.39e–3) and signaling by GPCR (p = 3.96e–4). Similar observation as pathway enrichment analysis could also be found in GO enrichment analysis (Additional file 1: Figure S2). In particular, TAP complexes, TAP-binding proteins (TAPBP), and MHC-I presentation (the translocation and peptide loading of this process are accomplished by TAP complexes and TAPBP) were highlighted with the introduction of the DE-based prioritization. In addition, 2-strategy winners indicated the significance of several pathways: type I diabetes mellitus (DM, p = 9.40e–4); antigen processing and presentation (p = 3.42e–5); TNFR2 signaling (p = 6.81e–3); and apoptosis pathway (REACTOME: apoptosis, p = 5.27e–3; PANTHER: apoptosis signaling pathway, p = 8.55e–3).

**Figure 6:**
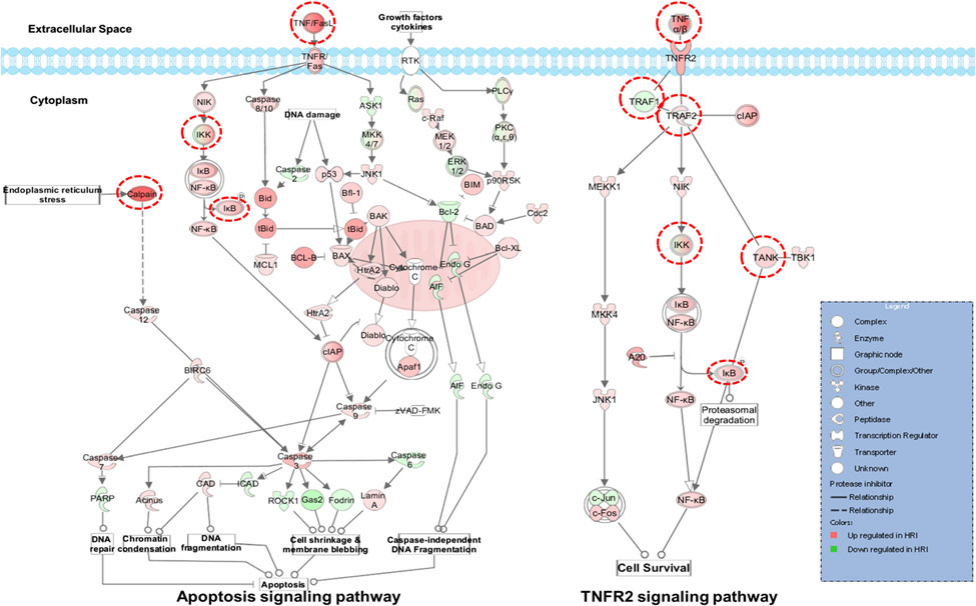
**Prioritized genes in apoptosis and TNFR2 signaling pathways.** The graphical representation of the pathways is generated by the ingenuity pathway analysis (IPA) tool. The prioritized genes were highlighted by red dotted circles. The apoptosis and TNFR2 signaling pathways were extracted from the “canonical pathway” mappings. Genes are color coded by their differential expression levels between resistance and susceptible mouse strains. In particular, the genes with higher expression in susceptible mice than in resistance mice were colored red; whereas those having lower expression in susceptible than resistance mice were shown in green. The symbols used to represent molecules and relationships were illustrated in the legend.

The two pathways highlighted by 2-strategy winners, namely, TNFR2 and apoptosis signaling pathways (Figure 6), share three top-ranked genes: *TNF*, conserved helix-loop-helix ubiquitous kinase (*CHUK*, also known as *IKK-α*), and nuclear factor of kappa light polypeptide gene enhancer in B-cells inhibitor-epsilon (*NF-IκBϵ*, also known as *IκBϵ*). Among these genes, the polymorphisms on *TNF* were reported to influence the severity of infection caused by H1N1 virus [36]. Moreover, the genetic polymorphism on *IκBϵ* is associated with invasive pneumococcal disease [56], a serious complication of seasonal and H1N1 influenza infection in 2009 [57]. These observations have suggested that the two pathways containing these genes may exert an important function in influenza host genetics. The results of the expression analysis in a previous study [33] (Supporting Information, File S4 in [33]) further showed that *TNF* is significantly upregulated (q-value = 1.98e–11) in severely infected mice compared with mildly infected mice, suggesting that the *TNF* expression is associated with the severity of host outcomes after influenza infection. Viral replication in lung epithelial cells is inhibited by TNF-*α*, and the virulence of H5N1 may be partly related to virus resistance to host TNF-*α*[58]. As such, anti-TNF can be administered to treat influenza infections [59]. However, the effectiveness of the TNF treatment remains controversial [60, 61]. The anti-TNF medicines demostrated efficacy in some patients but posed risk of increasing the severeity of influenza in others [62]. Faustman, *et al.*[63] have summarized the functions of TNF-mediated TNFR2 signaling pathway in autoimmune diseases and provided some information that may shed light on this perplexing question. For instance, systemic toxicity observed in some cancer patients receiving TNF treatment may be attributed to the widespread expression of TNFR1 in contrast to the limited distribution of TNFR2. TNF is a key signaling protein in the immune system [63] and can bind to two structurally distinct membrane receptors on target cells; these receptors are TNFR1 (also known as TNFRSF1A) and TNFR2 (also known as TNFRSF1B) [64], for diverse functions. In particular, TNF depends on TNFR1 in apoptosis; TNF also depends on TNRF2 to perform T-cell survival-related functions. The basis for anti-TNF medicines is to reduce the concentration of free TNF that can bind to functional T cells and lower the concentrations of TNFR2; as a result, TNF-mediated inflammation is reduced. Considering the relatively pervasive expression of TNFR1 compared with TNFR2, reduced TNF expression may play an even greater role in affecting the TNFR1-mediated apoptosis signaling pathway. Interestingly, the apoptosis signaling pathway was reported to play a role in ducks’ resistance (compared with chicken) to H5N1 infection [65]. We assumed that the high dose of anti-TNF medicines may significantly influence the process of T cell apoptosis in addition to the TNFR2 signaling pathway; hence, the delicate balance between TNF pro-survival and apoptotic effects is disrupted [66]. A TNFR2-specific agonist therapeutic strategy, however, would be a valid alternative treatment, given the limited distribution of TNFR2 [63]. Although few studies have been conducted to determine the exact functions of TNF in balancing the pro-survival effect and apoptosis during influenza infection, let alone the studies on investigating the possibility of applying TNFR2-specific antagonist in influenza treatment; we suggested that the relationship between apoptosis and TNFR2 signaling pathway would be a valuable topic in the field of influenza genetics study.

## Conclusions

Disease genes could be directly and efficiently predicted based on the prior knowledge of the biological processes involved in a particular disease. However, an alternative strategy, which could address the gaps left by the seed-based strategy, is needed when host genetics in resistance to influenza is partially understood and only a few known host resistance genes could be used as training set for the seed-based network strategy. In this study, we applied an integrated network analysis based on the known disease genes and DE levels between resistant/susceptible mouse strains. The DE-based strategy can overcome the inherent limitations of the seed-based strategy and complement the identification of promising candidates. In addition, the DE-based strategy can also add the credibility of a candidate gene for its role in host resistance to influenza to some extent. A list of genes suggested by multiple types of studies was specifically prioritized using the DE-based strategy. In our study, promising candidate genes supported by different types of evidence were significantly enriched in the 2-strategy winner set. Furthermore, top-ranked genes from both strategies indicated the significance of several biological processes and molecular functions. These results will enhance our understanding of the pathways associated with host genetic factors.

## Methods

### Candidate gene selection

We collected 17 chromosome regions (Table 2) that were reported as significantly or suggestively [logarithm of the odds (LOD) > 2.2)] associated with different traits related to influenza resistance from five independent genome-wide linkage studies. The human orthologs of the genes within the QTL regions were queried from Ensembl database (release 69) [67] by using the BioMart tool. A total of 876 conserved *Mus musculus* genes with human orthologs were obtained. Genes within different QTL regions formed separate candidate sets as input for the gene prioritization models. We assumed that at least one gene within each confirmed QTL region harbored variants associated with host susceptibilities.

### Network-based prioritization methods

To apply the network-based approaches, we evaluated several similarity measures between genes based on a protein-protein interaction network (STRING, version 9). STRING is a functional association network that contains associations inferred from various data sources (experimentally verified interaction, co-occurences in the literature, coexpression, and similar genomic context). The gene-gene interaction scores were extracted from the interaction scores between their corresponding protein products. When multiple proteins/isoforms are encoded by a single gene, all interactions will be considered if each encoded protein is linked to different proteins, or only the strongest interaction will be retained when some of the encoded proteins interact with the same protein. For the seed-based method, 14 genes (Table 1) related to different host responses to influenza virus infection were collected as seeds to construct our model. An initial score vector was constructed, in which the elements representing “seed genes” were given equal scores with sum of the probabilities equal to 1; whereas the scores for the other genes in the genome were initialized as 0. Four gene-gene similarity measurements were considered and evaluated in this step: DIR, SAR, RWR, and sHKDR. DIR ranks candidates according to the number of directly linked seed genes, whereas SAR uses the sum of association scores between a gene and the linked seeds in the STRING network. In RWR, the similarities between a gene and the seeds are assigned based on a steady-state probability vector, which is obtained after a number of iterative transitions from the current nodes to their randomly selected neighbors until convergence. sHKDR estimates the gene-gene similarities based on a diffusion kernel matrix, which is equivalent to a lazy random walker consisting of transitions from the current node to each of its neighbors with probability *β* and stay put with a probability of 1 - *d*_*i*_*β* (with *d*_*i*_ as the degree of node *i*) [17].

Rather than relying on prior knowledge of the disease, DE-based methods initialized scores for all genes in the network with the experimental data of the DE levels between susceptible and resistant hosts. Considering that very few public expression profiles for human subjects are currently available, we used a mouse expression profile (GSE30506) from the GEO database. This dataset consisted of 44 pre-CC mouse samples, among which 26 mouse lines showed severe (“**h**igh”) **r**esponse (**I**HC score: 4 or 5, % weight loss > 15%) to influenza virus **i**nfection (HRI mice), whereas 18 lines expressed mild (“**l**ow”) **r**esponse (**I**HC score: 0 or 1, % weight loss < 5%) to infection (LRI mice). The log2 ratio between the expression values of the HRI group to those of the LRI group was used as the DE measure. To investigate the effectiveness of the DE-based network method in identifying known host resistance genes, we used three methods: DER, DNR, and deHKDR. DER prioritizes candidates purely on their DE levels (represented as log2 ratio statistics) between susceptible and resistant hosts. DNR and deHKDR calculate a gene’s score by considering the DE levels of the gene and its surrounding neighbors. In particular, DNR applies equal weights for all neighbors; by comparison, deHKDR considers the initial interaction scores between the studied gene and its neighbors and applies the final weights from the heat kernel diffusion matrix. The mathematical details for each method were given in Additional file 1: Mathematical details of methods.

### Evaluation of model performance and screening of top-ranked genes

The performance of the seed-based network model was assessed by LOOCV test. In LOOCV, each seed gene is in turn removed from the training set and added to a set of 99 randomly selected genes from the whole genome. After prioritization was conducted based on a particular model, the rank of the seed genes among the 99 random genes reflects the discriminative ability of the model to identify host resistance genes. To quantify the enrichment of the seeds among the top-ranked genes, we calculated the proportion of the known genes that can be found at different rank thresholds (top 5%, 10%, 20%, etc.). Detection rates were then plotted against different rank thresholds, and the ROC curve was obtained. AUC was then used as a measure to assess the performance of a model. For DE methods, 11 seed genes were scored against 11*99 randomly selected genes. The ROC curve was then plotted. AUC was used to compare the effectiveness of different algorithms. We further tuned the required parameters to maximize the AUC for each method.

The top 10% candidates in a QTL candidate set prioritized by a method were termed as winners for that method, e.g., RWR winners were top-ranked genes by the RWR method. When a candidate gene was within multiple (overlapping) loci, each was counted as a separate prediction for a certain locus. Genes that were top ranked by both seed- and DE-based methods were referred to as 2-strategy winners.

### Literature annotation

Four types of studies related to host resistance or response to influenza, including genetic association studies [22, 27, 35–41], QTL studies [10, 14–16, 33], RNAi screenings [42–46], and microarray gene expression analyses [47–49], were collected and used to annotate the functional significance of these top-ranked genes. The genetic association studies were collected by conducting a literature search for the reported associations between gene variants and host resistance to influenza infection. QTL studies, in which the QTLs for candidate gene prioritization were collected, also provided a list of candidate genes based on independent evidence. In this study, supporting evidence from the genetic association studies and QTL studies was considered as genetic evidence. RNAi screenings [68] and microarray gene expression profiles [49] have also been extensively applied to identify host genes implicated in the life cycle of influenza virus and responses to virus infection. We also obtained the candidates recommended by these studies and referred to these types of supporting evidence as functional evidence. To accounting for the false positives in expression microarray, genes must be identified by at least two studies of expression analysis to be considered as supported. Additional file 2: Table S5 summarized the studies that provided supporting evidence including the criteria used to determine the candidates, number of identified genes, and corresponding references. Top ranked-genes suggested by multiple types of studies were summarized and listed in Table 3. To provide an overview of the functional significance of prioritized genes from seed- and DE-based network strategies, we grouped the top-ranked genes into 2-strategy winners (genes identified by both seed-based and DE-based strategy), DE-only winners (genes specifically identified by deHKDR method), and seed-only winners (genes specifically identified by seed-based strategy, either sHKDR or RWR). The proportions of top-ranked genes supported by genetic evidence and functional evidence or suggested by both types of evidence in each winner set were summarized and plotted as a stacked cylinder (Figure 4b). Using the prioritized genes as background, we evaluated the significance of the supported genes enrichment in each winner set by one-tailed hypergeometric test. The p value for each winner set was annotated above the corresponding cylinder (Figure 4b).

### Functional enrichment analysis

The BIOCARTA, KEGG, PANTHER, and REACTOME systems deposited by DAVID (version 6.7) [55] were applied in pathway enrichment analysis. GO and PANTHER were also used for gene ontology (including BP, MF and CC) enrichment analysis.

To reduce the redundancy from broad GO terms, we applied the GO FAT (GOTERM_BP_FAT, GOTERM_MF_FAT) categories, which screen out very broad GO terms based on the measured specificity of each term, in each top-ranked gene group (2-strategy, deHKDR, RWR, and sHKDR winners). In the PANTHER system, PANTHER_BP_ALL and PANTHER_MF_ALL were used for the gene set enrichment analysis. The enriched gene sets with p < 0.01 and FDR <0.25 were selected as significant sets. We classified all functional terms into four categories: pathway, biological process, molecular function, and cellular component. For each category, annotation terms that were significantly enriched in at least one winner group were illustrated as a heatmap. Each row in the heatmap denoted an enriched term, and each column represented a winner group. The cells were color coded using the fold enrichment of the annotation term by the corresponding winner group. All of the gene sets enriched by each method (2-strategy, deHKDR, RWR, and sHKDR) at a nominal significant level of p < 0.01, regardless of FDR, were listed in Additional file 3.

### Pathway analysis

We mapped top-ranked genes to the “canonical pathway” in ingenuity pathway analysis (IPA). The log2 ratios between the gene expressions of HRI mice and that of LRI mice were prepared as a dataset and imported into “Analyses, Datasets & Lists” OVERLAY in IPA for analysis. Genes with higher expression in HRI mice than in LRI were illustrated in red; otherwise, these genes were represented in green.

## Electronic supplementary material

Additional file 1: **Supplementary methods and results.** Additional methods and results referred to in the main text can be found here, including the mathematical details of seed-based (RWR, sHKDR, DIR, and SAR) and DE-based (deHKDR, DNR, DER) network methods. **Tables S1–S3.** show the parameter tuning for sHKDR (*β* and *m*), deHKDR (*β* and *m*), and DNR (*α*) method, respectively. Parameters that maximize the AUC of ROC for each method were selected in prioritizing candidate genes within mouse QTLs. **Figure S1.** shows the STRING sub-networks consisting of seed genes and their directly adjacent neighbors. The seed genes shown from panel (a) to (d) are: *C1qbp*, *H2-D1*, *Ifitm3*, and *Ifnar1*, respectively. The networks were visualized in Cytoscape [71] by using edge-weighted spring embedded layout. The distances between the seed and their neighbors are proportional to their interaction scores in STRING. Differential expression levels between resistant and susceptible mice were mapped onto each gene using node size and color shade as illustrated in the middle inset. All seed genes are highlighted using the same node size and bold fonts of their names. **Figure S2.** shows the heatmaps of GO enrichment for different winner groups. GO biological processes, molecular functions, and cellular components that are significantly enriched (p < 0.01 and FDR < 0.25) by 2-strategy, deHKDR, RWR, and sHKDR winners, are shown. The color intensity of each cell represents the fold enrichment of the corresponding winner group for each pathway. Only the significantly enriched pathways for each winner group are displayed. (PDF 2 MB)

Additional file 2: **Annotations of all top ranked genes. Table S4.** summarizs top-ranked genes by at least one method (deHKDR, RWR, and sHKDR). The following four types of supporting evidence for the functional role in influenza resistance were collected for each gene: genetic association, QTL, RNAi and gene expression studies. Immune related functional evidence from the annotations of RefSeq and UniProt databases or from literature is also noted. **Table S5.** summarizes the QTL studies, RNAi screenings, and gene expression analyses that were used to find supporing evidence. The methods used for candidate gene identification, the number of suggested candidates and corresponding reference for each study are shown. (XLS 102 KB)

Additional file 3: **Gene sets enriched for the prioritized genes.** All gene sets that were enriched by the prioritized genes at the nominal significance level (p < 0.01) are listed. For each enriched gene set, the table shows the number and the list of hit genes, total number of genes in the gene set, fold enrichment as compared with the genome background, and estimated FDR within each category. (XLS 108 KB)

## Abbreviations

QTL: Quantitative trait locus
eQTL: Expression quantitative trait locus
DIR: Direct interaction ranking
SAR: STRING association ranking
RWR: Random walk with restart
sHKDR: Seed-based heat kernel diffusion ranking
DER: Differential expression ranking
DNR: Direct neighborhood ranking
deHKDR: Differential expression-based heat kernel diffusion ranking
ROC: Receiver operating characteristic
AUC: Area under the curve
LOOCV: Leave-one-out cross validation
RNAi: RNA interference
CC: Collaborative Cross
HRI: High response to infection
LRI: Low response to infection.
