# Supplementary material for: Prioritizing genes responsible for host resistance to influenza using network approaches
Source: BMC Genomics. 2013 Nov 21;14(1):816. doi: 10.1186/1471-2164-14-816 (PMC4046670; doi:10.1186/1471-2164-14-816)
Supplement: Supplementary file 1 — Additional file 1: Supplementary methods and results. Additional methods and results referred to in the main text can be found here, including the mathematical details of seed-based (RWR, sHKDR, DIR, and SAR) and DE-based (deHKDR, DNR, DER) network methods. Tables S1–S3. show the parameter tuning for sHKDR (β and m), deHKDR (β and m), and DNR (α) method, respectively. Parameters that maximize the AUC of ROC for each method were selected in prioritizing candidate genes within mouse QTLs. Figure S1. shows the STRING sub-networks consisting of seed genes and their directly adjacent neighbors. The seed genes shown from panel (a) to (d) are: C1qbp, H2-D1, Ifitm3, and Ifnar1, respectively. The networks were visualized in Cytoscape [71] by using edge-weighted spring embedded layout. The distances between the seed and their neighbors are proportional to their interaction scores in STRING. Differential expression levels between resistant and susceptible mice were mapped onto each gene using node size and color shade as illustrated in the middle inset. All seed genes are highlighted using the same node size and bold fonts of their names. Figure S2. shows the heatmaps of GO enrichment for different winner groups. GO biological processes, molecular functions, and cellular components that are significantly enriched (p < 0.01 and FDR < 0.25) by 2-strategy, deHKDR, RWR, and sHKDR winners, are shown. The color intensity of each cell represents the fold enrichment of the corresponding winner group for each pathway. Only the significantly enriched pathways for each winner group are displayed. (PDF 2 MB) [file 12864_2013_5513_MOESM1_ESM.pdf]

Additional File 1

Prioritizing genes responsible for host resistance to influenza using network approaches

Suying Bao[1*], Xueya Zhou[2*], Liangcai Zhang[3], Jie Zhou[4], Kelvin Kai-Wang To[4], Liqiu Wang[5,6], Xuegong Zhang[2], You-Qiang Song[1§]

[1]Department of Biochemistry, The University of Hong Kong, Hong Kong, China

[2]Bioinformatics Division and Center for Synthetic and Systems Biology, TNLIST, MOE Key Lab of Bioinformatics / Department of Automation, Tsinghua University, Beijing, China

[3]Department of Biophysics, College of Bioinformatics Science and Technology, Harbin Medical University, Harbin, China

[4]Department of Microbiology, The University of Hong Kong, Hong Kong, China

[5]Department of Mechanical Engineering, The University of Hong Kong, Hong Kong

[6]Zhejiang Institute of Research and Innovation, The University of Hong Kong, Hong Kong

*These authors contributed equally to this work

[§]Corresponding author: YQS: songy@hku.hk

Mathematical details of methods

Seed-based prioritization methods

In the mathematical derivations below, we use a symmetrical adjacency matrix **A** to represent the gene network containing n genes. The entry $a_{ij} > 0$ if two genes i and j are linked with associated score of a_{ij}; otherwise, $a_{ij} = 0$. The seed-based methods rank genes according to their similarities (closeness) to seed genes in the gene-network. The similarity measure can be computed by the following two algorithms.

1) Random Walk with Restart (RWR)

The random walk on networks is defined as an iterative transition from the current node to a randomly selected neighbor [1]. To obtain the transition probability, Tong suggested using the following normalization of the adjacency matrix [2]:

$$\tilde{\mathbf{A}} = \mathbf{D}^{-1/2}\mathbf{A}\mathbf{D}^{-1/2} \qquad (1)$$

where **D** is an $n \times n$ diagonal matrix, with $d_{i,i} = \sum_{j=1}^{n} a_{ij}$.

The random walk method was applied to the gene prioritization problem by Köhler *et al.* [3] with the modification to allow restart from source nodes with probability r (we used $r = 0.5$ as suggested by the author). It is defined by

$$\mathbf{p}^{t+1} = (1-r)\tilde{\mathbf{A}}\mathbf{p}^{t} + r\mathbf{p}^{0} \qquad (2)$$

where $\mathbf{p}^t$ is a vector with i-th element p_i^t representing the probability of being at node i at step t. The vectors is initialized with $p_i^0 = \frac{1}{s}$ if node i is one of the s seed genes; and $p_i^0 = 0$ otherwise. This enables random walk to start from each seed gene with

equal probabilities. After enough steps, random walk will enter the steady-state when the probability of being at each node become stabilized. We then rank the candidates according to their values in the steady-state probability vector $\mathbf{p}^{\infty}$. It can be approximated by iterating (1) until the probability vector converges:

$$\sum_{i=1}^{n}\left|p_i^{t+1} - p_i^{t}\right| < 10^{-6}.$$

2) Seed-based Heat Kernel Diffusion Ranking (sHKDR)

The heat kernel diffusion matrix was introduced by Chung *et al.* [4] defined as $\mathbf{H} = e^{- \;\mathbf{L}}$, where β is a diffusion factor controlling the magnitude of the diffusion. And matrix $\mathbf{L}$ is the normalized Laplacian of the graph defined as $\mathbf{L} = \mathbf{I} - \mathbf{D}^{-1}\mathbf{A}$, where $\mathbf{I}$ is the identity matrix, $\mathbf{A}$ and $\mathbf{D}$ were defined as above. The kernel diffusion matrix computes the similarity of two nodes in the network as the probability of reaching one node at some time point after a random walk starting from another node. To simplify the computation, here we used the discrete approximation introduced by Yang et al. [5].

$$\mathbf{H} = \left(\mathbf{I} + \frac{-}{N}\mathbf{L}\right)^{N} \tag{3}$$

with N being the number of iterations. The candidate genes are ranked by their average similarities to seeds:

$$score(j) = \frac{1}{s}\sum_{i \in \text{seeds}} H_{ij} \tag{4}$$

We tried several sets of parameters of (N, β) in cross-validation studies to evaluate their performances.

3) Direct Interaction Ranking (DIR) and STRING Association Ranking (SAR)

To demonstrate the advantage of similarity measures that take both direct and indirect links into account, we also used two methods that only consider direct interaction partners for comparison: Direct Interaction Ranking (DIR) [6] and STRING Association Ranking (SAR). In the DIR method, the candidate genes are ranked by the number of directly linked seed genes. To implement the method, we only used links with scores greater than 0.4 which represents the “medium” confidence in STRING database. In SAR method, the candidate genes are ranked by the sum of interaction scores (weights) with their direct neighbors.

Differential expression (DE)-based prioritization methods

1) Differential expression-based Heat Kernel Diffusion Ranking (deHKDR)

This method was first proposed by Nitsch et al [7] to prioritize genes for diseases with little prior knowledge about their underlying genes, and extended in a subsequent study [8]. Recall the scoring function for the seed-based HKDR(4). It can be alternatively expressed using a vector form as

$$\mathbf{p}_{\sim} = \mathbf{p}_0 \mathbf{H} \qquad (5)$$

where $\mathbf{p}_0$ stands for a preference vector, which is initialized the same way as the probability vector in seed-based HKDR. The final score for each candidate gene can be found in $\mathbf{p}_\alpha$. DE-based HKDR method has the same weighting scheme as equation (5), but instead of relying on seed genes, $\mathbf{p}_0$ is initialized by the differential expression levels comparing the cases and controls. In this way, each candidate gene is scored by a weighted sum of DE levels of itself and its neighbors, with the weights for neighbor

genes equaling to their similarities to the candidate.

The assumption behind this approach is that the true disease genes tend to be surrounded by differentially expressed neighbors. We tested if the assumption is valid for the seed genes when evaluating the performance of the method. We also tried several sets of parameters to fine tune the performance on predicting known host resistance genes.

2) Direct Neighborhood Ranking (DNR)

As a comparison, we also implemented a network analysis method of differential expression based on Direct Neighborhood Ranking (DNR) matrix. In this method, neighbors of a candidate gene *i* are defined as all genes directly linked to the candidate in the network with confidence score $a_{ij} > \pounds$. Here we set ε = 0.15 as suggested by Nitsch [8]. The candidate gene *i* is scored by

$$\hat{x}_i = \sim \cdot x_i + \frac{1-\sim}{N} \sum_{j \neq i,\, j: \sim_{ij} > \pounds} x_j \tag{6}$$

where x_i is the differential expression level of gene *i*. The parameter *α* controls the contribution of a candidate gene's own expression to its score, which is named as steady factor relative to the diffusion factor in heat kernel diffusion models. *N* denotes the number of direct neighbors near candidate gene *i*.

Supplementary Tables

Table S1 - Parameter optimization for β and *m* in seed-based Heat Kernel Diffusion Ranking model

m = 1	AUC of sHKDR	β = 0.5	AUC of sHKDR
β = 0.25	0.904	*m* = 1	0.912
β = 0.5	0.912	*m* = 2	0.895
β = 0.75	0.907	*m* = 3	0.906
β = 1	0.890	*m* = 5	0.903

Table S2 - Parameter optimization for β and *m* in the differential expression-based Heat Kernel Diffusion Ranking model

m = 1	AUC of deHKDR	β = 0.5	AUC of deHKDR
β = 0.25	0.869	*m* = 1	0.915
β = 0.5	0.887	*m* = 2	0.897
β = 0.75	0.915	*m* = 3	0.891
β = 1	0.908	*m* = 5	0.888

Table S3 - Parameter optimization for α in the Direct Neighborhood Ranking model

α	AUC of DNR
α = 0.75	0.845
α = 0.5	0.864
α = 0.25	0.854
α = 0	0.829

Supplementary Figures

Supplementary Figure S1: The STRING sub-networks consisting of a single seed gene and its directly adjacent neighbors

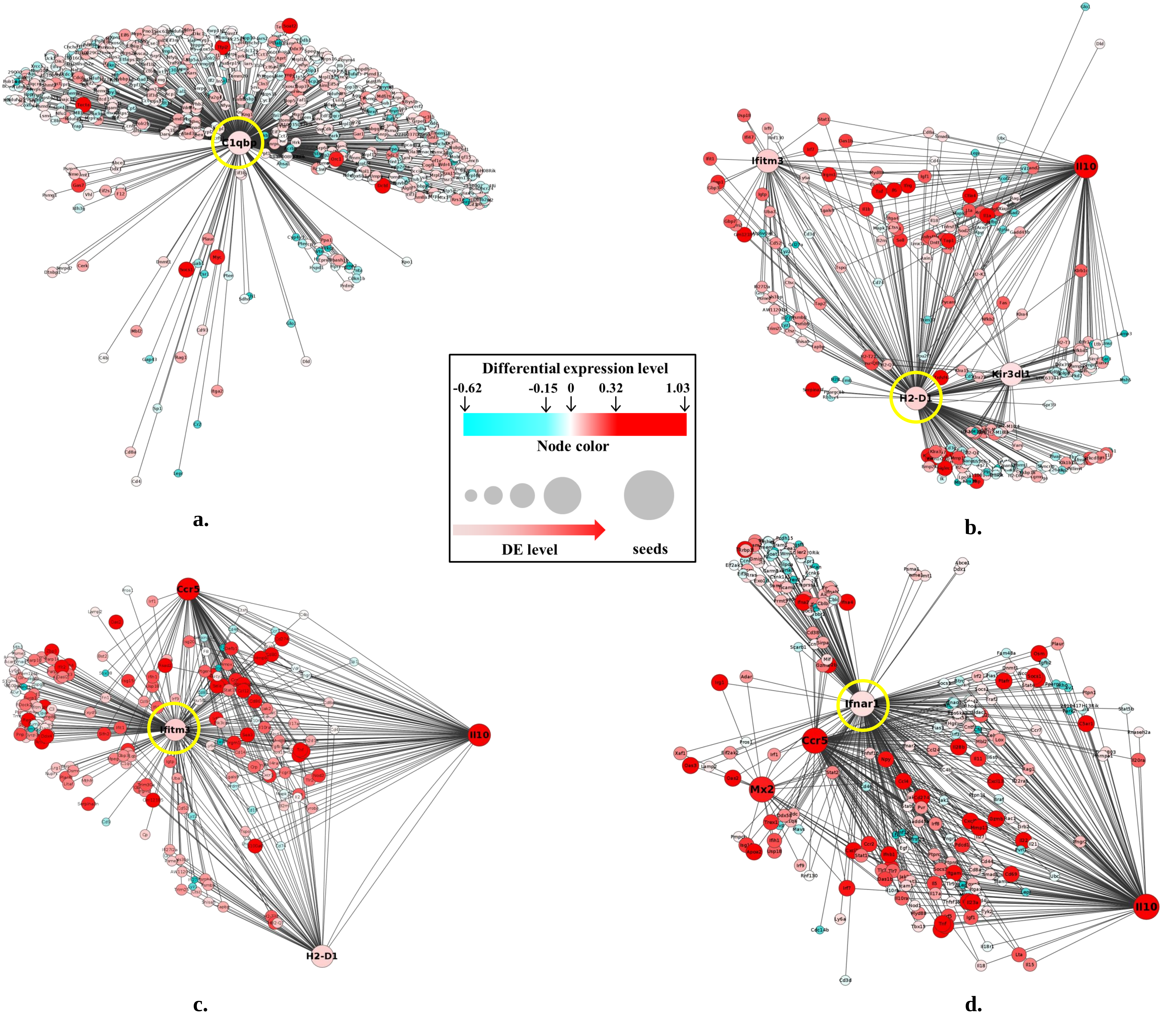

Supplementary Figure S2: Heatmaps of functional enrichment for different winner groups

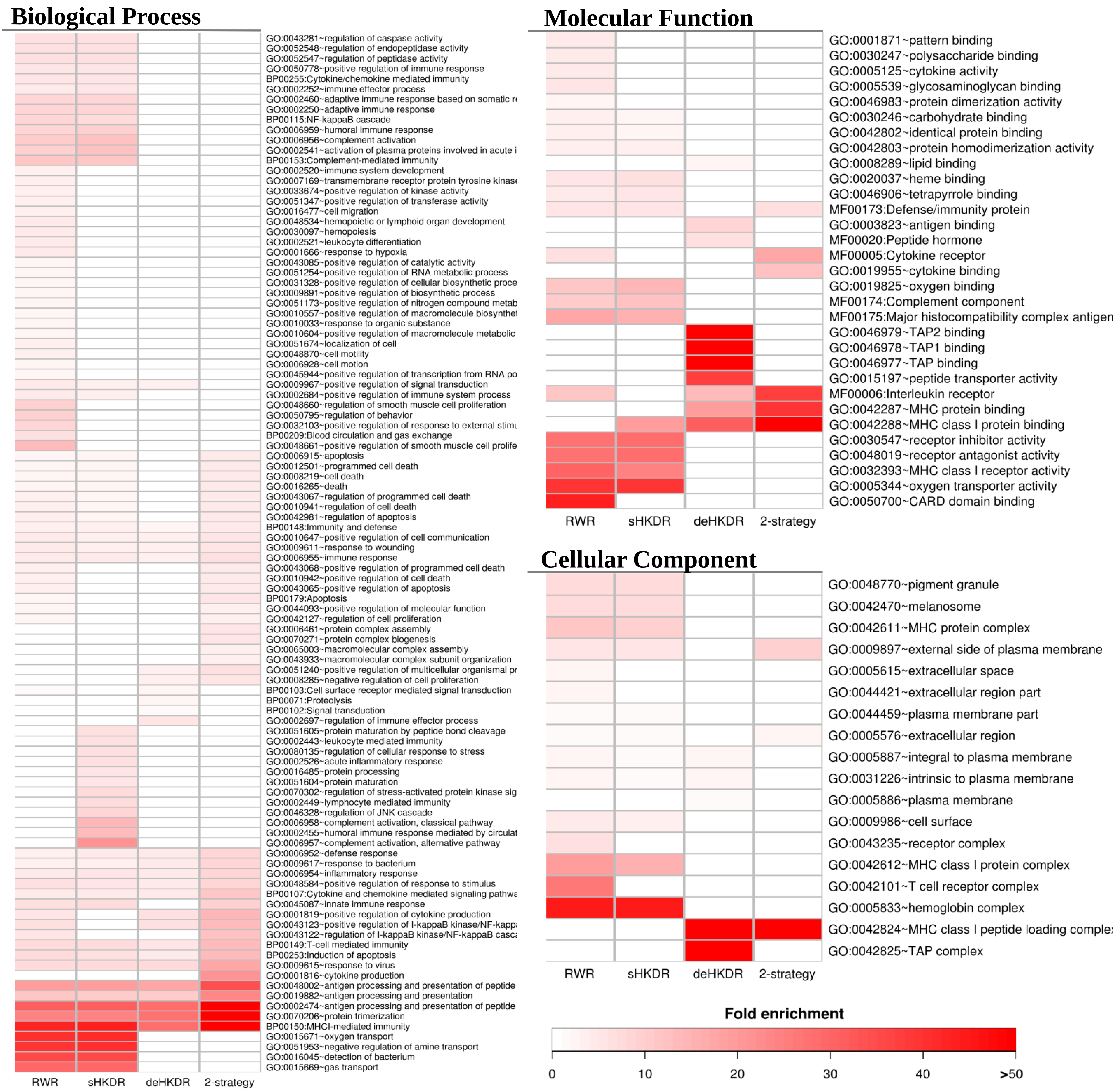

Reference

1. Can T, Çamoğlu, O., and Singh, A.K: **Analysis of protein-protein interaction networks using random walk**. In: *In BIOKDD '05: Proceedings of the 5th international workshop on Bioinformatics: 2005; New York, USA*: Association for Computing Machinery; 2005.
2. Tong HH, Faloutsos C, Pan JY: **Random walk with restart: fast solutions and applications**. *Knowl Inf Syst* 2008, **14**(3):327-346.
3. Kohler S, Bauer S, Horn D, Robinson PN: **Walking the interactome for prioritization of candidate disease genes**. *American journal of human genetics* 2008, **82**(4):949-958.
4. Chung F YS: **Coverings, heat kernels and spanning trees**. *Electron J Comb* 1999.
5. Yang H KI, Lyu MR: **Diffusion rank: a possible penicillin for web spamming**. In: *30th annual international ACM SIGIR conference on Research and development in information retrieval: 2007; Amsterdam*: ACM; 2007.
6. Oti M, Snel B, Huynen MA, Brunner HG: **Predicting disease genes using protein-protein interactions**. *J Med Genet* 2006, **43**(8).
7. Nitsch D, Tranchevent LC, Thienpont B, Thorrez L, Van Esch H, Devriendt K, Moreau Y: **Network analysis of differential expression for the identification of disease-causing genes**. *PloS one* 2009, **4**(5):e5526.
8. Nitsch D, Goncalves JP, Ojeda F, de Moor B, Moreau Y: **Candidate gene prioritization by network analysis of differential expression using machine learning approaches**. *BMC bioinformatics* 2010, **11**:460.
